# Understanding lactation policies and resources across a university system: survey and document review

**DOI:** 10.1186/s12884-024-06541-9

**Published:** 2024-05-15

**Authors:** Emily L. Ashby, Sritha Donepudi, Heather M. Padilla

**Affiliations:** University of Georgia, Athens, GA USA

**Keywords:** Pregnancy, Breastfeeding, Lactation, Maternity, Workplace, Pumping, Support, Workplace facilities, Employer support

## Abstract

**Background:**

In the U.S., employees often return to work within 8–12 weeks of giving birth, therefore, it is critical that workplaces provide support for employees combining breastfeeding and work. The Affordable Care Act requires any organization with more than 50 employees to provide a space other than a restroom to express breastmilk and a reasonable amount of time during the workday to do so. States and worksites differ in the implementation of ACA requirements and may or may not provide additional support for employees combining breastfeeding and work. The purpose of this study was to conduct an analysis of the policies and resources available at 26 institutions within a state university system to support breastfeeding when employees return to work after giving birth.

**Methods:**

Survey data was collected from Well-being Liaisons in the human resources departments at each institution. In addition, we conducted a document review of policies and online materials at each institution. We used univariate statistics to summarize survey results and an inductive and deductive thematic analysis to analyze institutional resources available on websites and in policies provided by the liaisons.

**Results:**

A total of 18 (65.3%) liaisons participated in the study and revealed an overall lack of familiarity with the policies in place and inconsistencies in the resources offered to breastfeeding employees across the university system. Only half of the participating liaisons reported a formal breastfeeding policy was in place on their campus. From the document review, six major themes were identified: placing the burden on employees, describing pregnancy or postpartum as a “disability,” having a university-specific policy, inclusion of break times for breastfeeding, supervisor responsibility, and information on lactation policies.

**Conclusion:**

The review of each institution’s online resources confirmed the survey findings and highlighted the burden placed on employees to discover the available resources and advocate for their needs. This paper provides insight into how institutions support breastfeeding employees and provides implications on strategies to develop policies at universities to improve breastfeeding access for working parents.

## Introduction

### Breastfeeding & return to work

Exclusive breastfeeding for the first six months of an infant’s life is one of the greatest protective health factors for both the breastfeeding parent and child [1]. Parents who breastfeed have lower incidence of hypertension, type 2 diabetes, and ovarian and breast cancer,infants who receive breastmilk have greater sensory and cognitive development, as well as protection from multiple acute and chronic illnesses [2]. More specifically, infants who were not breastfed had increased incidence of illnesses such as leukemia, asthma, other respiratory outcomes, and immunity components [3]. Even after adjusting for parental age, education, smoking status, race, gender, birth weight and order, and other factors, there was still 1.3 times higher risk of infant mortality in infants who were formula fed as opposed to breastfed [4]. Despite the benefits of breastfeeding, only 58.3% of infants born in the US in 2017 were breastfed at six months old, and only a quarter of those infants were exclusively breastfed [1].

The CDC, WHO, and American Academy of Pediatrics agree that the best practices for breastfeeding include exclusive breastfeeding for the first six months of life [1, 2]. The availability of time off from work postpartum, and perceived workplace support, was positively associated with higher rates of exclusive breastfeeding for longer periods of time [5]. Of the 41 member nations in the Organization for Economic Cooperation and Development (OECD), 40 mandate paid leave for mothers (and sometimes fathers, too) postpartum [6]. The only nation within OCED that does not provide federal paid leave is the United States [6]. In the United States, new parents often return to work within the first three months of giving birth due to lack of paid parental leave. Lack of paid time off impacts the ability to sustain breastfeeding, as seen in breastfeeding rates dropping from 81% at childbirth to 67% at 6 weeks, 49% at 12 weeks, and 33% at 6 months postpartum [7]. Women who hold professional jobs or return to work within the first 6 months postpartum are more likely to stop breastfeeding early, which justifies an examination of how employer policies affect ability to breastfeed [7]. To support employees in achieving breastfeeding goals while returning to work, it is imperative for workplaces to have clear, informative, and supportive policies in place that set the standard for all employees and their supervisors. Previous research suggests that workplace employee health policies that help protect, and support employee health have lower rates of absenteeism and higher productivity [8].

### Impact of workplace policies

The workplace has a major influence on employee health and behavior; workers in the US spend an average of 8.42 h at work each workday [9]. Comprehensive employee wellness programs have been shown to be effective in promoting healthy employee behaviors and reducing employer healthcare costs [10]. Policies are an important element of comprehensive employee wellness programs [11]. Worksite policies set the written expectation of the value of health, thereby creating a culture of prioritizing wellbeing at the worksite, and specifying the available resources provided to employees to promote health [12]. The Workplace Health in America (WHA) survey (2019) found that nearly half (46.1%) of worksites offered some type of health promotion or employee wellness program, with a majority focusing on physical activity or nutrition programming. Only 7.6% of worksites offered lactation support programs, the lowest of all program categories [13].

### The case for lactation policies

While a significantly personal decision, the decision to breastfeed can be impacted by the policies, resources, and culture at the workplace. A workplace culture that encourages positive perceptions of breastfeeding children among employees and supporting adequate time and space for pumping breastmilk has been associated with parents sustaining breastfeeding after returning to work [8, 14]. Breastfeeding self-efficacy for female employees was positively associated with female coworker support of breastfeeding [15]. Programs focused on lactation support policy at the organizational and managerial level in the workplace have been successful in facilitating continued exclusive breastfeeding rates among employees [16]. Further, creating designated spaces for pumping breastmilk at work and maintaining a clean and comfortable environment within those spaces has been shown to increase employee rates of continued breastfeeding after returning to work [17].

### Impact of the affordable care act

The Affordable Care Act requires any organization with more than 50 employees to provide a space other than a restroom and a reasonable amount of time during the workday for women to express breastmilk [18]. Each state within the United States applied this federal mandate differently, allowing employers to interpret the law in various ways [18]. When the stipulations of the ACA were applied in a comprehensive workplace policy there were positive associations with better breastfeeding outcomes among employees [19]. Women who were provided both adequate break time and private space were 2.3 times more likely to be breastfeeding exclusively at six months and 1.5 times more likely to continue breastfeeding exclusively with each passing month compared with women without access to these accommodations [20]. Additionally, human resource officers and those charged with overseeing and directing employee wellness policies have a major impact on breastfeeding policy creation and implementation within their worksite [21].

### Barriers in lactation policy

Despite some positive changes in national laws surrounding lactation support at work, implementation and success of lactation policies face difficulties. Barriers for workplaces introducing lactation policies include limited resources, perceptions on the need for breastfeeding policies, and certain organizational characteristics such as workforce age [22]. Additionally, even in worksites where breastfeeding support initiatives exist, employees encounter challenges in gaining equitable access to the resources that may be available via their workplace policies [23]. Even in female-dominated professions, a lack of female-supportive language within breastfeeding policies creates a culture of apathy and does not encourage translation of policy to practice, decreasing the potential impact of these policies on breastfeeding outcomes [24].

### Present study

To better understand the implementation of breastfeeding policies in workplaces, we conducted a study with 26 institutions that are part of a state university system with a system-wide employee well-being program. Each institution has its own leadership and employee policies and practices. More specifically, we analyzed the differences in breastfeeding policies and resources available on each campus when employees return to work after giving birth.

## Methods

### Setting & participants

This study was conducted within a southeastern university system comprised of 26 institutions that range from four-year Research One universities to smaller community colleges. The institutions within the system ranged in total number of employees from 141 to 10,544. Characteristics of the institutions are described in Table 1. Within each institution, the human resources department identified one staff person to be the “Well-being Liaison.” The Well-being Liaisons were tasked with providing wellness content, resources, and communication of wellbeing policies to all sectors of their colleges and universities. They served populations including faculty, staff, facilities management, and every person employed by the institution. The well-being liaison connected the institution to the system-wide well-being program and was responsible for connecting its campus to well-being efforts, events, and information. Collectively, the liaisons were part of a comprehensive approach to create a culture and environment of well-being. The 26 individuals serving as well-being liaisons at the time of this study were invited to participate.

**Table 1:** Institution employee demographics

Institution	Total Faculty	% Female Faculty	% Black Faculty	% Hispanic Faculty	Full Time Employees
Institution 1	1054	44.1	6.1	5.2	4096
Institution 2	1014	26.6	2.7	5.2	6834
Institution 3	1515	50.8	14.7	3.4	4090
Institution 4	1842	39.3	5.5	3.6	9091
Institution 5	956	51.3	7.1	4.0	2273
Institution 6	1209	48.2	9.3	3.9	2237
Institution 7	433	58.2	7.6	3.7	1240
Institution 8	382	52.1	7.1	3.7	748
Institution 9	175	54.9	50.9	1.1	529
Institution 10	204	55.9	27.9	2.9	439
Institution 11	280	47.1	11.1	4.3	584
Institution 12	92	45.7	58.7	1.1	488
Institution 13	293	53.9	7.2	4.4	687
Institution 14	115	53.0	1.7	2.6	183
Institution 15	252	50.8	12.7	2.8	427
Institution 16	122	40.2	41.0	3.3	486
Institution 17	660	48.9	4.7	5.2	1054
Institution 18	135	50.4	7.4	1.5	265
Institution 19	31	41.9	71.0	3.2	167
Institution 20	94	54.3	9.6	4.3	187
Institution 21	140	54.3	5.0	1.4	197
Institution 22	54	50.0	5.6	5.6	158
Institution 23	444	52.7	10.6	4.1	403
Institution 24	120	57.5	10.0	1.7	226
Institution 25	86	52.3	14.0	0.0	210
Institution 26	62	53.2	3.2	3.2	133

### Recruitment

Data collection occurred from September 2021—April 2022. An initial email was sent to all well-being liaisons inviting them to complete an online survey in Qualtrics. Follow-up emails were sent three weeks after the initial invitation. Due to low response rate after reminder emails, the research team adapted the survey to a phone interview and two members of the research team called any liaison who had not responded to the initial surveys and invited them to participate. Reaching out to participants via phone occurred in three phases. In the first phase, one member of the research team called each liaison inviting them to participate. For those who did not answer the initial phone call, a second member of the research team called back. Any remaining institutions that were not reached with the first two phone call attempts were called a third and final time by the first research team member. Upon completion of the survey, all participants were mailed an incentive valued at $20.

### Liaison survey

The liaison survey was adapted from the Breastfeeding and Employment Study organization survey [25]. This survey aimed to gain insight into the liaisons’ perspectives and knowledge regarding lactation policies and resources on their respective campuses. The information gathered was based on their individual observations and knowledge of institution policies. The survey was organized into four categories of breastfeeding support: policy, resources, lactation space availability, and time. Examples of the survey questions in each category are provided below. All survey questions are provided in Table 3 with corresponding response options.

### Policy

The survey contained twelve questions regarding university-specific policies on breastfeeding at work and maternity leave. For example, we asked “Does your institution have a written policy on breastfeeding and/or pumping milk at work?”. We also asked about employee awareness of policies (e.g., “Are all employees informed of this policy?” Response options were “yes” and “no”. Additional questions inquired about parental leave payment policies. For example, we asked “Where does that maternity leave pay funding come from?” Respondents could select multiple options including “short-term disability insurance,” “accrued time,” “organizational-paid leave,” and write in an institutional-specific source of funding. This section of the survey also offered the opportunity for each liaison to provide their formal, written policies on breastfeeding and maternity or parental leave.

### Resources

This section of the survey contained questions on resources available to employees on campus, including whether the resource was available and the provider of the resource. Seven resources were assessed: access to a lactation consultant, educational classes on breastfeeding/pumping, electric breast pumps or pumping kits, and onsite daycare availability. For each resource, respondents indicated if the resource existed and whether it was provided by the wellness program, employee assistance program, health insurance or other.

### Lactation spaces

The next section of the survey included questions focused on pumping breastmilk at work. We asked seven questions about specific places on campus where employees can express breastmilk. An example question was “Are restrooms the only spaces available for breastfeeding or pumping?” with answer choices of “yes” or “no.” We also asked about availability of designated lactation spaces and the amenities available in these spaces. The questions we asked included, “Is there any space(s) on your campus dedicated solely to breastfeeding or pumping breastmilk?” and, “What amenities are available in these spaces?” The first question had response options of “yes” or “no,” and the second question had a list of possible amenities including a sink, electrical outlet, and refrigerator.

### Time

The last section of the survey included questions regarding time provided to employees for breastfeeding, as well as what maternity leave looked like at each institution. We asked five questions inquiring to the availability and qualification for maternity leave as well as time provided at work for pumping. An example of these questions included, “Are employees who qualify for Family and Medical Leave Act (FMLA), eligible to receive pay during maternity leave?” and “Does your organization allow women to extend maternity leave past 12 weeks?” Answer choices to these questions included “yes,” “no,” or “I don’t know.” We also asked about non FMLA eligible employees by asking each liaison, “How much time can employees who are NOT eligible for FMLA take off work for maternity leave?” Possible answers included “Less than 2 weeks,” “2–6 weeks,” “7–12 weeks,” or “Other.”” We then asked questions about time to pump once employees returned to work. Questions included “What are, if any, the flexible time scheduling options available to full-time employees at your college?” with response options of flextime, telecommuting, job sharing, or none. Additionally, we asked about time given to pump during the workday (e.g., “When are women allowed to pump breastmilk during work?” with answer options of only during break or lunch times, as needed, or unsure.

### Document review

During the liaison survey, we asked each participant to provide copies of their institution’s breastfeeding and maternity leave policies. The document review included policies we received from the liaisons and policies or information found on institution websites. Two research team members reviewed each institution’s website for information on specific employee breastfeeding policies, lactation rooms on campus, and any other information relevant for parents returning to work after giving birth. These resources included employee handbooks, student handbooks, publicly available online policies and any other information that could be found on human resource webpages.

### Data collection

All liaison survey data were collected and stored via Qualtrics. Initially the survey was self-administered but due to the low response rate, the survey was adapted to allow the survey to be conducted via phone by trained research staff. No changes were made to the main aspects of the survey questions when adapted to phone administration. Instead, the wording shifted from “Please note how women are made aware of lactation policies on campus” on the electronic survey to “Can you describe how female employees are made aware of lactation policies on your campus?” on the phone-adapted version. Research team members would then list the possible answer choices that matched those on the electronic survey. Data from the document review were organized in a Microsoft Excel spreadsheet.

### Analysis

Survey data were analyzed by univariate statistics to provide frequencies and means. Data from the document review was analyzed by constant comparative method to develop themes via a deductive and inductive approach [26].

### Deductive approach

Each policy was reviewed based on its compliance with and expansion of the requirements for employers through the Affordable Care Act [18]. Compliance was defined by the research team as the University providing a policy that stated, at minimum, a space other than a restroom on campus for employees to express breastmilk as well as time during the workday to express breastmilk. The ACA amended the Fair Labor Standards Act requiring employers to provide “reasonable break time for an employee to express breastmilk for her nursing child for one year after the child’s birth each time such employee has need to express the milk” ([18] #115). Employers are also required to provide “a place, other than a bathroom, that is shielded from view and free from intrusion from coworkers and the public, which may be used by an employee to express breastmilk” ([18] #115). Based on language found within institution policies, the research team coded the data as compliant or not compliant with the ACA requirements.

### Inductive approach

We used an inductive approach to allow themes to emerge from the written policies available at each institution. Two research team members read through each available policy independently to identify recurring themes and organized the institutions based on the findings. After independently identifying themes, the two research team members met to review their findings and confirm the categories identified. All discrepancies in findings were resolved during the second meeting.

## Results

The survey was sent to 26 well-being liaisons. Nine well-being liaisons completed the self-administered version of the survey, and nine participants completed the survey by telephone. The remaining eight liaisons either declined to participate or were not reached via telephone after the third attempt. The total response rate for surveys was 69.2% (*n* = 18).

### Demographics

Survey participants were mostly white (70.6%), non-Hispanic (94.4%), female (88.9%), with some postgraduate education (41.2%). Most participants held the wellness liaison position for nine years or less (78.9%). Table 2 describes the sample demographics.

**Table 2:** Liaison demographics

Variable	Participants (*N* = 18)
	(no %)
Sex	
Male	2 (11.1%)
Female	16 (88.9%)
Hispanic/ Latino	
Yes	1 (5.6%)
No	17 (94.4%)
Age (*n* = 16)	
20–29	1 (6.3%)
30–39	7 (43.7%)
40–49	4 (25%)
50–59	2 (12.5%)
60–69	2 (12.5%)
Race (*n* = 17)	
Black or African American	5 (29.4%)
White	12 (70.6%)
Highest Education Level (*n* = 17)	
Some college of technical/ vocational training	1 (5.9%)
Associate’s degree (2 years)	4 (23.5%)
Bachelor’s degree (4 years)	5 (29.4%)
Postgraduate Work (PhD, or MD)	7 (41.2%)

### Liaison survey

Overall, many of the well-being liaisons were uncertain about the existence of breastfeeding policies on their campus beyond the federal requirements of the Affordable Care Act (i.e., providing a space to pump other than a restroom and reasonable break time during the workday to pump). The results of the survey are described in detail in Table 3.

**Table 3:** Summary of survey questions & response options

Survey section	Survey questions	Response options
Policy	Does your institution have a written policy on breastfeeding and/or pumping milk at work?	Yes (1) No (2) I don't know (3)
	Are all employees informed about this policy (e.g., when hired, at orientation, HR website)?	Yes (1) No (2) I don't know (3)
	How are employees informed about the lactation policy? There can be multiple answers to this question!	HR website available to public (1) HR intranet available to employees (2) Orientation (3) Hiring packet (4) Emails (5) Other (6)
	Are only pregnant women informed?	Yes (1) No (2) I don't know (3)
	Where does that maternity leave pay funding come from?	Short term disability insurance (1) Accrued time (sick, vacation, personal) (2) Donated time (bank, other employee) (3) Organization paid leave (other than disability, accrued, or donated time) (4)
	Is that extended maternity leave funded through…	Short term disability insurance (1) Accrued time (sick, vacation, personal) (2) Donated time (bank, other employee) (3) Organization paid leave (other than disability, accrued, or donated time) (4)
	Is their job held for women on maternity leave until their return?	Yes, for all employees (1) Yes, only for employees with FMLA leave (2) I don't know (3)
	Does your college prohibit women from bringing their infants to and from work for breastfeeding?	Yes (1) No (2) Explain if necessary (3)
	In what ways are **employees** made **aware** of available lactation services or breastfeeding support on campus?	Organization policies/employee handbook (1) Human resources-website/newsletters (2) Health Insurance/Open Enrollment (3) Employee orientation or meetings/in-services (4) Informal/word of mouth (5) Employees seeking services or resources (6) Other (please specify): (7)
	In what ways are supervisors made aware of available lactation services or breastfeeding support on campus?	Organization Policies/Employee Handbook (1) Human Resources-Website/Newsletters (2) Health Insurance/Open Enrollment (3) Manager Orientation or Meetings/In-Services (4) Informal/Word of Mouth (5) Employees seeking use of benefits (6) Other (please specify): (7) Supervisors are not made aware of lactation services or breastfeeding support (8)
	How are supervisors are made aware of benefits other than lactation services (i.e., flexible scheduling options, family-friendly benefits, and wellness benefits)?	Organization Policies/Employee Handbook (1) Human Resources-Website/Newsletters (2) Health Insurance/Open Enrollment (3) Manager Orientation or Meetings/In-Services (4) Informal/Word of Mouth (5) Employees seeking use of benefits (6) Other (please specify): (7)
	Does your college allow women to express/pump milk at work?	Yes (1) No (2) I don't know (3)
Time	Are employees who qualify for Family and Medical Leave Act (FMLA), eligible to receive pay during maternity leave?	Yes (1) No (2) I don't know (3)
	When are women allowed to pump breast milk during work?	Only during set break times and/ or lunch time (1) As needed (2) I don't know (3)
	How much time can employees who are NOT eligible for FMLA take off work for maternity leave?	Less than 2 weeks (1) 2–6 weeks (2) 7–12 weeks (3) Other (4)
	Does your organization allow women to extend maternity leave past 12 weeks?	Yes (1) No (2) I don't know (3)
	What are, if any, the flexible time scheduling options available to full-time employees at your college?	Part-time returning (1) Flextime (2) Telecommuting (3) Job sharing (4) None of these are made available (5)
Lactation Spaces	Are the restrooms the only spaces available for breastfeeding or pumping milk on your campus?	Yes (1) No (2) I don't know (3)
	Is there any designated space where women are allowed to breastfeed or pump milk when not being used for other purposes (e.g., a conference room)?	Yes (1) No (2) I don't know (3)
	What are the spaces where women are allowed to breastfeed or pump breast milk on your campus?	Own offices (1) Vacant offices (2) Conference rooms (3) Lactation spaces (4)
	Is there any space(s) on your campus dedicated solely to breastfeeding or pumping breast milk?	Yes (1) No (2) I don't know (3)
	What amenities are available in these spaces? (select all that apply)	Locking door (1) Sink (2) Electrical outlet (3) Refrigerator (to store breast milk) (4) No amenities are available (5)
Resources	Using the matric table, please indicate which of the following possible breastfeeding resources are available at your college. For each resource, please indicate how it is made available to employees	List of resources: Access to a lactation consultant (either onsite or through a referral service) (1) Employee support group (2) Educational classes that include information on breastfeeding and pumping (3) Educational materials/ handouts on breastfeeding or pumping (Please provide copies of any materials if possible) (4) Electric breast pump (5) Kit for pumping (6) Onsite daycare (7) List of resource sources: N/A (1) Wellness Program (2 Employee Assistance Program (3) Health Insurance (4) Family Friendly Benefits (5) Other Organizational benefits (6)

### Policy

Half of the participants said there is no formal policy (50%) for breastfeeding at work on their campus, and 58.3% of those indicated that not all employees are informed of the policy. Supervisors are made aware of the lactation resources available mostly when employees are seeking use of those benefits (42.1%), by the employee handbook (31.6%), the HR website (31.6%), or by simple word of mouth (31.6%).

### Resources

The survey assessed specific resources listed as best practices from the Breastfeeding and Employment Study [25] including providing breast pumps, educational materials, educational classes on breastfeeding, access to a lactation consultant, on-campus childcare, and a pumping kit to all breastfeeding employees. All resources, except for childcare services, were reportedly available to individuals who are enrolled in health insurance. Resources such as providing breast pumps to employees, educational materials, educational classes, or access to a lactation consultant were not provided through any on-campus program. Less than half of institutions (47.4%) reported offering onsite daycare for employees’ children.

### Lactation spaces

Most liaisons indicated that they believe their institution allows employees to pump milk at work (86.7%) and 53.8% of liaisons responded employees can pump as needed. Most participants noted that there are specific locations other than restrooms available for pumping (64.3%). However, only 42.1% mentioned lactation-specific spaces available to everyone. The majority of liaisons said that lactating employees can only access their own private offices (if their position provides it), a vacant office, or a vacant conference room.

### Time

Most institutions have a formal leave policy under FMLA (93.3%), which provides 12 weeks of unpaid, job-protected leave per year for those who qualify. Funding for leave for pregnant and postpartum employees predominantly comes from accrued time (i.e., sick leave; 68.4%), short-term disability benefits (57.9%), and donated time (only available at select places; 42.1%). Most participating liaisons (75%) noted that job protection and holding are only available to employees who qualify for FMLA, which may not cover all workers employed across a variety of positions on their campuses. Flextime was the most common scheduling option offered to employees returning to work postpartum (35.7%) with telecommuting as the second most common option (28.6). Most liaisons indicated that lactating employees could pump at work as needed (53.9%) though 31% said employees can pump only during set break times. Table 3 below summarizes the survey questions and results.

### Document review

Using a deductive approach first, results show that most participating universities had policies or statements that contained language matching the Affordable Care Act, such as space other than a restroom for breastfeeding and reasonable break times. This approached was used as a baseline for understanding if and how these institutions have integrated this federal mandate into their individual campus policies and employee handbooks.

Overall, six themes emerged from the policies during the inductive approach of the document review. The themes included placing the burden on breastfeeding employees, describing pregnancy or postpartum as a “disability,” having a university-specific policy, inclusion of break times for breastfeeding, supervisor responsibility, and information on lactation policies. Definitions of each theme are provided in Table 4.

**Table 4:** Summary of document review

Theme	Definition	No. of Institutions
Placing Burden on Breastfeeding Employees	Institution policy contains no information on lactation OR responsibility for accessing the resource falls solely on the employee	13
Disability Language	Referring to “disability due to pregnancy” and funding maternity leave from disability leave	3
Break Times	Policy clearly states providing break time for employees to breastfeed	10
University Specific Policy	The institution has created and made available an institutional lactation policy that is different from the general requirements of the ACA or university system	13
Supervisor Responsibility	Explicit mention of supervisors’ involvement in providing break time and resources to employees returning to work post-partum	5
Lactation Spaces	Policy contains information about lactation spaces on campus	11

### Placing the burden on breastfeeding employees

Thirteen institutions had no policy or lacked information on breastfeeding on their websites or within their employee handbooks. Additionally, thirteen institutions who had policies in place lacked information about the supervisors’ role in supporting breastfeeding for employees or suggested that it was the employees’ responsibility to identify resources. Below is an example of language within a policy that placed the burden on the individual to navigate lactation around their work schedule when they return to work postpartum:

“If the employee uses time other than a regularly scheduled break time [for pumping breastmilk] or if the employee’s department does not have designated break times, then the employee will be required to either: (1) make up that time during the same workday; (2) use vacation leave; or (3) take leave without pay for that period of time.”

OR

“If employees request accommodations for breastfeeding, units should contact Office of Human Resources regarding their responsibilities in accordance with federal and state regulations.”

### Disability language

Three institutions had policies that referred to “disability due to pregnancy” or classified any policies regarding pregnancy or breastfeeding within their disability category. Almost all institutions only provided paid leave for employees via short-term disability leave. Still, there was no distinction between leave due to pregnancy/ postpartum and other situations that would be classified as disability. An example of this theme was the following statement in an institution’s parental leave policy, “Disability due to pregnancy shall be considered as any other disability…”.

### Break times

Ten institutions included language for break times specific to “nursing,” “pumping,” or “lactation” within their policies. Example quotes of this language include:

“Lactating mothers shall be granted flexible and reasonable breaks, using their normal break periods and mealtimes, to accommodate milk expression.”

“[Employees will be provided] …reasonable break times to express milk for her baby, so long as the break does not unduly disrupt the operations of the college. The break time shall, if possible, run concurrently with any break time already provided to the employee. No work duties will be required to be performed during this break period.”

### University-specific policy

Half (*n* = 13) of the universities had their own institutional policy on breastfeeding and lactation. The remaining institutions (*n* = 13) only referred to the larger university system policy or had no information. Institutions that created individual breastfeeding policies contained language such as:

“The University supports parents employed by [this institution] by providing lactation and nursing support. A lactation and nursing parent support program allows a lactating parent to express breastmilk periodically during the workday or nurse an infant child. This policy is in accordance with the Official Code of [State], Federal Labor Standards Act, as well as [University System], [this institution], local, State and Federal regulations.”

### Supervisor responsibility

Policies at five institutions contained information regarding supervisor responsibility. For each of these schools, there is explicit mention of female employees needing to coordinate with their direct supervisor to ensure reasonable break times for breastfeeding are met. Examples of this language within policies included:

“Lactating mothers who wish to express milk during the work period should keep supervisors informed of their needs so that appropriate accommodations can be made to satisfy the needs of both the employee and the department.”

“Supervisors should attempt to provide as much schedule flexibility and break time as reasonably possible to accommodate the employee’s needs.”

### Lactation spaces

Twelve (46.15%) institution policies contained language about lactation spaces on campus. Of those 12, very few provided specific details on where the spaces are, how to access them, and what amenities were available. An example of language of a policy that included details is provided here:

“The designated lactation room is located in [location]. The lactation room provides an electrical outlet, comfortable chair, and nearby access to hot running water and soap.”

Some institutions mentioned having a designated lactation space on campus, but there was no information on who to contact or how to access the space. Instead, there was vague language about including a space other than a restroom, but no further details on a lactation space on campus. An example of this vague language includes, “[This institution] will provide to any employee who is breastfeeding her child…”.

## Discussion

The purpose of this project was to understand how a group of institutions in higher education support breastfeeding employees through policies and resources when they return to work. With the passage of the Affordable Care Act in 2014, all worksites that employ more than 50 people are required to provide a place other than a bathroom and reasonable break time during work hours for employees to pump breastmilk [18]. Understanding how this policy has or has not been translated into worksites provides insight into how organizations are caring for their employee’s health and well-being.

### Summary of findings

Our survey of Well-being Liaisons within a university system revealed a large variation in lactation policies and resources across institutions. While we found that liaisons reported and formal policies reflected the minimum ACA requirements, how these policies are implemented on campuses was unclear. Of great concern is that about half of institutions did not have a formal policy for breastfeeding. Further, communication of the policy to supervisors was very passive in nature and only when needed for an employee or through written material available to all employees. Establishing policies at all institutions and making all supervisors aware of the policy increases the likelihood that employees are supported in their breastfeeding goals when they return to work.

Although the survey of liaisons suggested there is opportunity for employees to pump on campus, without any formal policy or a lack of detail in existing policies, the burden is placed on employees to discover and advocate for their needs. The lack of explicit language protecting breastfeeding employees leads to discrepancies in experiences when returning to work. Previous studies in workplace wellness have highlighted the difficulty in adopting wellness policies, especially across worksites of varying size and cultures [17, 20, 22].

Except for onsite daycare, best practice resources for breastfeeding were available to employees, though these resources were available through an individual’s insurance provider. Even within institutions where childcare may be provided, no information was provided on the capacity of those childcare settings or the difficulties an employee may experience enrolling their child. This may mean that employees on spouse’s insurance plans or those who are on spouse’s insurance plans or those who are ineligible for insurance (i.e., part-time, or temporary employees) do not have access to these resources. Lactation space and time are included explicitly in the ACA language and seem to be available at all institutions. However, the details of how those amenities are provided to ensure all employees have equitable access to both space and time to pump are not detailed in the policies. More research is needed to understand why these discrepancies exist across institutions, how to combat them, and how to support complex work structures like colleges and universities in adopting these across institutions exist, how to combat them, and how to support complex work structures like colleges and universities in adopting these kinds of policies. The review of available policies from the institutions corroborates the findings from the survey of the liaisons that there is a lack of detail around the practice of providing support for breastfeeding when employees return to work after giving birth.

As of the time of this data collection, in the U.S., the only national job protection for parents returning to work after giving birth was through FMLA, which is not specific to pregnancy but covers any medical condition. As such, most of the policies described pregnancy as a disability and, in some cases, individuals’ leave from work and return to work after birth was treated as any other medical condition.

The majority of the language in the policies reviewed and that used in the workplace has historically referred to women, female employees, and maternity leave. While this language is evolving to be more inclusive of all parents, we used the terms consistent with the policy to avoid any assumptions that the policies may apply more broadly to all parents. More research is needed to determine how these policies affect parents who do not identify as women and those who adopt a child.

It was not the intent of this research to compare policies and resources available between institutions who had differing racial and ethnic profiles. However, because some institutions had more Black and Hispanic faculty, we did a post-hoc analysis to see if there was variation in policies and resources between those institutions and institutions with majority white faculty. We did not find any differences among institutions employing large numbers of Black and Hispanic faculty relative to those with majority white faculty. However, this is an area that should be further investigated in a larger sample.

Since data collection for this project occurred, US legislation has moved forward to create further protections for breastfeeding employees. In December 2022, the Providing Urgent Maternal Protections (PUMP) Act was passed, and it went into effect on April 28, 2023, [27]. The PUMP Act placed stronger regulations on the Affordable Care Act’s provision of “reasonable break time” for a pumping employee and supports the employee’s ability to pursue legal actions against an employer not abiding by this new law [27]. More research is needed to understand if the PUMP Act improves implementation of policies and practices that allow employees to express breast milk. This study provides a baseline status of policies in a higher education setting prior to implementation of the PUMP Act.

### Limitations

This study is not without its limitations. First, due to limited response to surveys sent via email, the survey was adapted to be conducted over the phone thus, word change occurred. Also, the individuals who serve as the Well-being Liaisons from each institution do not all have the same job title or training as this is not their primary role in the workplace. Therefore, some participants may have been more familiar with the policies than others depending on their regular job duties. Previously, all Well-being liaisons participated in a well-being board, but from initial data collection to the present, that group has been disbanded. Although they all work on separate campuses across the university system, it is possible that participants at the time of data collection communicated with one another about the survey, introducing potential response bias to survey results. However, no indication was made during the phone interviews that any participants had heard about the study or had spoken to other liaisons. Furthermore, the authors reviewed these findings in the context of how they apply to pregnant employees who gave birth and are the primary or sole breastfeeding partners. There is a need for more LGBTQIA + representation in breastfeeding literature, and future research in this field should highlight the unique experiences of employees who induce lactation for an adopted child or a partner’s child.

Additionally, the authors recognize the reality of potential social bias being introduced due to the phone medium for some survey responses versus electronic medium for others. While our survey and document review investigated the availability of childcare on campus, the questions did not investigate whether or not employees could visit the on-campus childcare and breastfeed their child. Finally, due to access restrictions, the document review was limited to online information that the research team could access or that which was provided by the liaisons. More nuances and detailed information that may have been available on each institution’s policies were unavailable to this research team.

### Strengths of study

Despite these limitations, this current study has some strengths. We included two sources of data – Well-being Liaison surveys and qualitative document review. This allows us to compare data from both sources to understand the policies and practices at the institutions. To the author’s knowledge, the scope of research done on what policies and resources exist for breastfeeding employees within complex work structures is limited. Investigating the differences across a university system sheds light on inconsistencies in how employees experience returning to work after giving birth and breastfeeding.

### Implications

Academic institutions like the colleges and universities analyzed in this study employ a wide range of personnel. Due to the complexity of their employment and work structure, it is difficult to assess how institutions support all their faculty and staff. The findings from this current study highlight the need for improved policies that support employees when returning to work postpartum. Creating consistent, written policies across a university system and allowing individual institutions to adapt them is necessary. To create a healthier work environment for employees working at colleges and universities, university leadership and human resource officials need to establish policies and resources to ease employees’ return to work postpartum.

## Data Availability

The datasets used and/or analyzed during the current study are available from the corresponding author on reasonable request.

## Abbreviations

ACA: Affordable Care Act
FMLA: Family Medical Leave Act
